# Organic Anion and Cation SLC22 “Drug” Transporter (Oat1, Oat3, and Oct1) Regulation during Development and Maturation of the Kidney Proximal Tubule

**DOI:** 10.1371/journal.pone.0040796

**Published:** 2012-07-13

**Authors:** Thomas F. Gallegos, Gleb Martovetsky, Valentina Kouznetsova, Kevin T. Bush, Sanjay K. Nigam

**Affiliations:** 1 Department of Pediatrics, University of California at San Diego, La Jolla, California, United States of America; 2 Department of Biomedical Sciences, University of California at San Diego, La Jolla, California, United States of America; 3 Department of Medicine, University of California at San Diego, La Jolla, California, United States of America; 4 Department of Cellular and Molecular Medicine, University of California at San Diego, La Jolla, California, United States of America; National Cancer Institute, United States of America

## Abstract

Proper physiological function in the pre- and post-natal proximal tubule of the kidney depends upon the acquisition of selective permeability, apical-basolateral epithelial polarity and the expression of key transporters, including those involved in metabolite, toxin and drug handling. Particularly important are the SLC22 family of transporters, including the organic anion transporters Oat1 (originally identified as NKT) and Oat3 as well as the organic cation transporter Oct1. In ex vivo cultures of metanephric mesenchyme (MM; the embryonic progenitor tissue of the nephron) Oat function was evident before completion of nephron segmentation and corresponded with the maturation of tight junctions as measured biochemically by detergent extractability of the tight junction protein, ZO-1. Examination of available time series microarray data sets in the context of development and differentiation of the proximal tubule (derived from both in vivo and in vitro/ex vivo developing nephrons) allowed for correlation of gene expression data to biochemically and functionally defined states of development. This bioinformatic analysis yielded a network of genes with connectivity biased toward Hnf4α (but including Hnf1α, hyaluronic acid-CD44, and notch pathways). Intriguingly, the Oat1 and Oat3 genes were found to have strong temporal co-expression with Hnf4α in the cultured MM supporting the notion of some connection between the transporters and this transcription factor. Taken together with the ChIP-qPCR finding that Hnf4α occupies Oat1, Oat3, and Oct1 proximal promoters in the in vivo differentiating rat kidney, the data suggest a network of genes with Hnf4α at its center plays a role in regulating the terminal differentiation and capacity for drug and toxin handling by the nascent proximal tubule of the kidney.

## Introduction

The proximal tubule of the nephron plays a key role in solute reabsorption from the glomerular filtrate in the kidney. In addition, proximal tubule cells have the capacity for basolateral uptake of certain organic compounds from surrounding capillaries, which are subsequently catabolized or apically exported into the tubular lumen for excretion. To carry out these transport functions, the polarized epithelial cells comprising the proximal tubule must establish tight junctions that prohibit paracellular diffusion, thus allowing active transport of small molecules and ions between fluid compartments (i.e., blood and urine) in a regulated, transcellular fashion. In this way the kidney is capable of regulating the serum concentration of a large number of plasma solutes. Critical proximal tubule transporters are members of the solute carrier (SLC) family of transporters, including transporters of both organic anions (OATs) and organic cations (OCTs). Because many potentially nephrotoxic endogenous metabolites, exogenous toxins and widely-administered pharmaceuticals are organic anions, the proper development of OAT function has great bearing on kidney function and clinical outcome under normal and pathophysiological conditions [1], [2]. Among the OATs, Oat1 (originally identified as NKT1) [3], [4] and Oat3, are present on the basolateral membrane of the proximal tubule and are believed to comprise the rate-limiting step in the in vivo renal clearance of organic anion drugs, metabolites and toxins from the blood [5], [6], [7], [8], [9]. They may play a broader physiological role in an hypothesized remote sensing and signaling network of multispecific SLC and ABC “drug” transporters [10], [11]. Moreover, a recent report by the International Drug Transport Consortium has emphasized the clinical importance of these transporters in renal drug handling [12].

In rodents, the kidney begins to develop around mid-gestation, and the nephrons develop toward the end of gestation, with further nephrogenesis and differentiation occurring throughout the first several weeks of life [13], [14]. In humans, the development of the metanephric kidney is initiated around 5 weeks post implantation. The kidney gains rudimentary function about a month later, and is considered to be structurally complete around 32 weeks of gestation [15]. Nevertheless, the fundamental process of nephrogenesis leading to proximal tubule functionality appears largely similar at the cellular and molecular level in humans and rodents. Nephron maturation may be incomplete in certain premature deliveries, which is believed to render the pre-term infant susceptible to immediate drug toxicity, in addition to the implications for long-term compromised kidney function and a possibly increased predisposition for nephrotoxicity during adulthood [16].

The epithelial nephron is developmentally derived from the metanephric mesenchyme portion of the intermediate mesoderm. The collecting system to which the nephron attaches, also derived from the intermediate mesoderm, develops from the Wolffian duct-derived ureteric bud. The nephron begins to develop at the tips of the ureteric bud, when the surrounding capping mesenchyme receives a UB-derived signal to undergo nephrogenesis. Cells of the MM form a condensate and undergo a transition to epithelium. The epithelial cells undergo morphological changes, starting with the formation of a renal vesicle, followed by the comma and then the S-shaped bodies. The S-shaped body elongates and further differentiates into the nephron, including the proximal and distal segments, the loop of Henle, the epithelial portion of the glomerulus, and the connecting segment attached to the collecting system.

The mechanisms regulating the process of proximal tubule differentiation (including postnatal maturation) remain incompletely defined. For example, it has been established that early in proximal tubule development (i.e. before s-shaped body), conditional deletion of Notch 2 or downstream notch effector Rbp-j in MM cells results in the specific disruption of proximal fate [17]. In contrast, inhibition of gamma-secretase (a proteolytic enzyme modulating canonical Notch-signaling) at different stages of kidney development revealed that blocking Notch activity at the S-shaped body stage did not prevent proximal tubule formation, suggesting that proximal cell fate is already specified at that stage [17]. Here we have attempted to further elucidate genetic interactions key to proximal tubule maturation, especially as they pertain to directional transport of drugs, toxins, and metabolites. Analysis of time series microarray data, characterizing proximal tubule development, together with time series biochemical analyses of tight junction maturation and functional analysis organic anion transport, implicated Hnf4α in these processes and correlated well with developmental expression patterns of Oat1, Oat3 and Oct1. Using chromatin immunoprecipitation followed by qPCR, Hnf4α was found to be able to regulate the expression of the SLC22 “drug” transporters Oat1, Oat3, and Oct1 during kidney proximal tubule maturation.

## Materials and Methods

### Tissue Culture

Metanephric mesenchyme (MM) and spinal cord tissues were dissected from e13 rat tissue. The MM was separated from the ureteric bud (UB) and cultured as described before [18]. Cultures were grown on 0.4 micron Trans-well filters over DME-F12 with 10% FBS and 1% antibiotics, and incubated at 37°C with 5% CO_2_ and 100% humidity. All animal procedures were approved by IACUC.

### Microarray Data Analysis

Microarray data for the s-shaped body and proximal tubule, published previously [19], [20], were downloaded from the GenitoUrinary Database Molecular Anatomy Project (GUDMAP) database website (www.gudmap.org). The data were analyzed using Genespring GX 11.5.1 (Agilent). Comparisons were made by T-test, using a FDR of 0.05. The in vivo and ex vivo gene lists were compared (6768 in vivo and 650 ex vivo genes), and the intersecting genes (108 by Entrez gene ID) were used to build a network diagram using Ingenuity Pathways Analysis (IPA). The core of the network is a number of genes that are known to contribute to either the development of the nephron toward proximal tubule or are indicative of the mature functional tubule itself: NOTCH1 [21], NOTCH2 [17], RBPJ (recombination signal binding protein for immunoglobulin kappa J region) [17], JAG1 (jagged 1), DLL1 (delta-like 1) [17], HEY1 (hairy/enhancer-of-split related with YRPW motif 1) [22], HES1 (hairy and enhancer of split 1) [22], [23], HNF1A (hepatocyte nuclear factor 1 alpha) [24], HNF4A (hepatocyte nuclear factor 4 alpha) [25], Hyaluronic Acid [26], CD44 (hyaluronate binding protein) [26], HMMR (hyaluronan mediated motility receptor) [26], CDH1 (e-cadherin) [27], TJP1 (tight junction protein 1) [27], WT1 (Wilms tumor 1) [28], LHX1 (LIM homeobox 1) [29], POU3F3 (POU class 3 homeobox 3) [30], SLC22A6 (organic anion transporter 1) [12], SLC22A8 (organic anion transporter 3) [12], SLC22A1 (organic cation transporter 1) [12], SLC22A2 (organic cation transporter 2) [13], SLC47A1 (multidrug and toxin extrusion protein 1; MATE1) [13], SLC15A2 (H+/peptide transporter) [13]. Transcription factor binding site prediction was done using MatInspector (Genomatix) [31] using the inputs: Oct1: GXP_277944, GXP_258878, GXP_420738; Oat1: GXP_50197, GXP_278073, GXP_304691; Oat3: GXP_278139, GXP_304585, GXP_50241. Candidates were constrained by raising positional matrix similarity threshold to the maximum stringency, and requiring detection in at least 8 out of 9 transcripts. SOMs were generated using Gene Expression Dynamics Investigator (GEDI) after normalizing the highest probeset expression data from the sample averages across the four conditions [32]. NMF was performed using GenePattern using the default conditions [33].

### Immunofluorescence

Tissues were briefly rinsed in PBS and fixed in 4% paraformaldehyde. Samples were rinsed in PBST with 0.1 M Glycine, and incubated in TBST at room temperature for one hour. Samples were blocked with 2% Bovine serum albumin in TBST for one hour, then incubated with primary antibody (E-cadherin 1∶1000 or ZO-1 whole supernatant), washed in PBS, and then in blocking buffer containing secondary antibody at 1∶1000 (Alexafluor 488 or Alexafluor 594, Invitrogen, Carlsbad, CA). The samples were mounted under Fluoromount, and imaged using a Nikon D-Eclipse C1 fluorescent microscope.

### Detergent Extractions and Western Blotting

Tissues were solubilized using CSK-1 buffer [34]. The lysate was centrifuged at 15,000 rpm at 4°C for 15 min. The supernatant was reserved and the pellet was re-suspended. The samples were diluted to 1× in Invitrogen NuPAGE LDS sample buffer with dithiothreitol, and run on Invitrogen 10% precast gels. The proteins were transferred to a nitrocellulose membrane, blocked, and probed using an antibody for GRP94 (1∶1000) from Invitrogen or ZO-1 (provided by Dan Goodenough, Harvard). Proteins were visualized using horseradish peroxidase conjugated secondary antibodies and Supersignal West Pico chemiluminescent substrate (Pierce, Rockford, IL).

### Organic Anion Uptake

The assay was completed as described previously [18]. Briefly, samples were washed with PBS, and incubated in PBS containing 6-carboxy fluorescein alone (1 µM) or with probenecid (1 mM). The samples were washed on ice with cold PBS, mounted on slides under PBS and imaged on a confocal microscope (Nikon D-Eclipse C1).

### Chromatin Immunoprecipitation and QPCR

Chromatin was prepared using whole kidneys from approximately 2-week-old (P13) Sprague Dawley rats. Kidneys were flash-frozen in liquid nitrogen for future processing. Thawed tissue was minced in ice-cold PBS containing 1% formaldehyde, followed by a thirty minute incubation with rotation at room temperature. Fixation was quenched with glycine, followed by homogenization with a tissue grinder, and several washes with PBS containing 0.5% Nonidet P-40. To isolate nuclei, the cells were disrupted using a glass Dounce homogenizer. Chromatin was then fragmented using a Cole-Parmer handheld microtip sonicator.

To quantify the chromatin, inputs were treated first with RNAse and then with Proteinase K, followed by de-crosslinking overnight at 65°C, phenol extraction, and ethanol precipitation. For qPCR, melt curves were inspected to ensure specificity and signals were normalized to input DNA to account for primer efficiency. Primer Pair Sequences (5' to 3'): Untr1 tgttgcctttttgcttttcac, taaccagcgtgctttgtgac; Untr4 ccatctctagccacagcatct, gctttccctccatgacactc; Slc22a8 prom (−362) cagaagcaccgctctcatc, tcgagtctgagggtccagag; Slc22a6 prom (−225) agcagaccctgaaagctgag, tttctctctccctcggttct; Slc22a1 prom (−149) gccctctccctggtatcac, actggggtggcttgaaatc.

## Results

**Figure 1:**
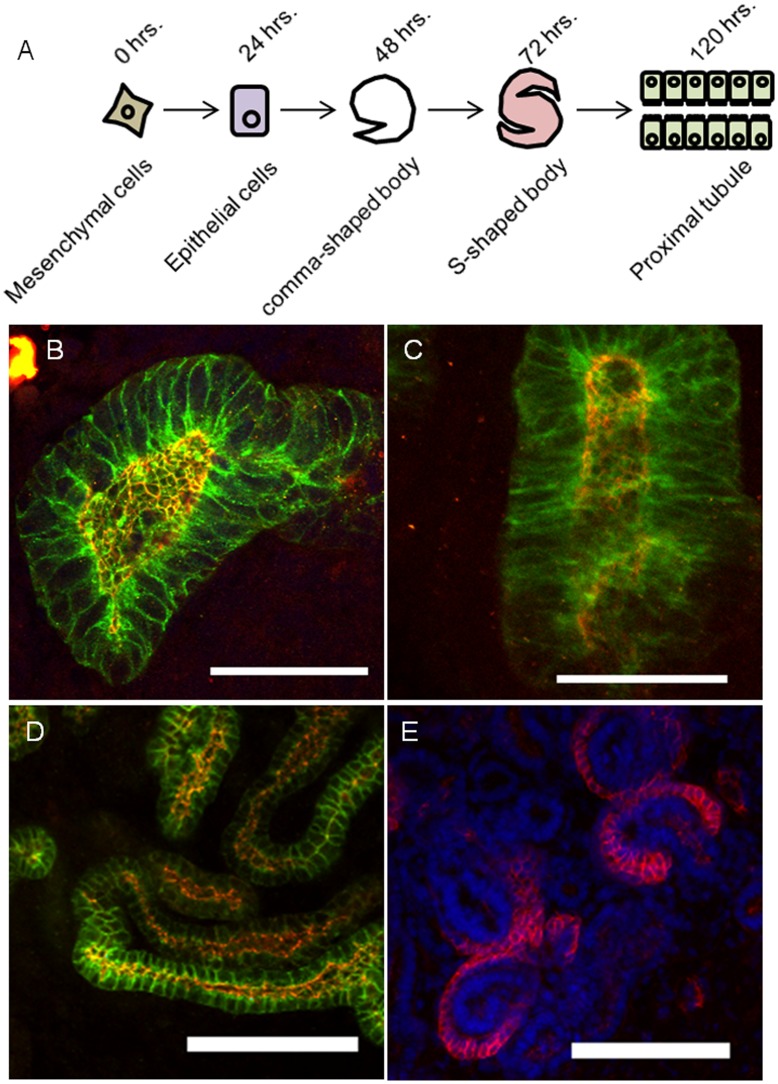
Development of epithelial characteristics and nephron segmentation. (A) A diagram showing the morphological progression of nephron development ex vivo, and the corresponding culture time. B) Immunofluorescent image of a 2 day (early) nephron culture. The tight junction marker ZO-1 is shown in red, and the adherens junction and marker E-cadherin is shown in green (panels B-D). C) Immunofluorescent image of 3 day (early) nephron culture. D) Immunofluorescent image of the convoluted late tubule (5 days and beyond). E) Immunofluorescent image of a 5 day (late) nephron culture. Peanut lectin marks developing podocytes (red), DAPI (blue). The late nephron begins segment specific differentiation. Bar is 50 µm (B, C, E) or 100 µm (D).

The proximal tubule develops from the metanephric mesenchyme (MM), one of the two primordial tissues of the metanephric kidney. Nephrons form within the MM in response to signals arising from the ureteric bud (UB), in a process that can be largely recapitulated in culture using heterologous tissues or soluble factors as inducers [35], [36]. The ex vivo culture of nephrons from isolated MM allows for analysis of roughly synchronized development on a morphogenetic scale. Cultured MM will undergo many of the morphogenetic changes seen in the in vivo developing kidney, including mesenchymal to epithelial transition, followed by tubular morphogenesis through comma and S-shaped bodies, then through tubular elongation and differentiation into the different segments of the epithelial nephron (see diagram in Figure 1A) [37].

Immunofluorescent microscopic examination of the comma-shaped bodies clearly shows groups of cells expressing E-Cadherin at the lateral membrane, with concentrations at the apico-basolateral junctions of the cells (Figure 1B). Similar to in vivo development, there appears to be a well-defined lumen demarcated by staining for the tight junction marker, ZO-1. At three days, tubulogenesis occurs (Figure 1C). Late cultures (beyond 3 days) develop into long tubules (Figure 1D), with islands of peanut lectin-positive cells (marker of the podocyte lineage) at the ends of some tubules (Figure 1E) as differentiation is allowed to continue (∼5 days of culture).

**Figure 2:**
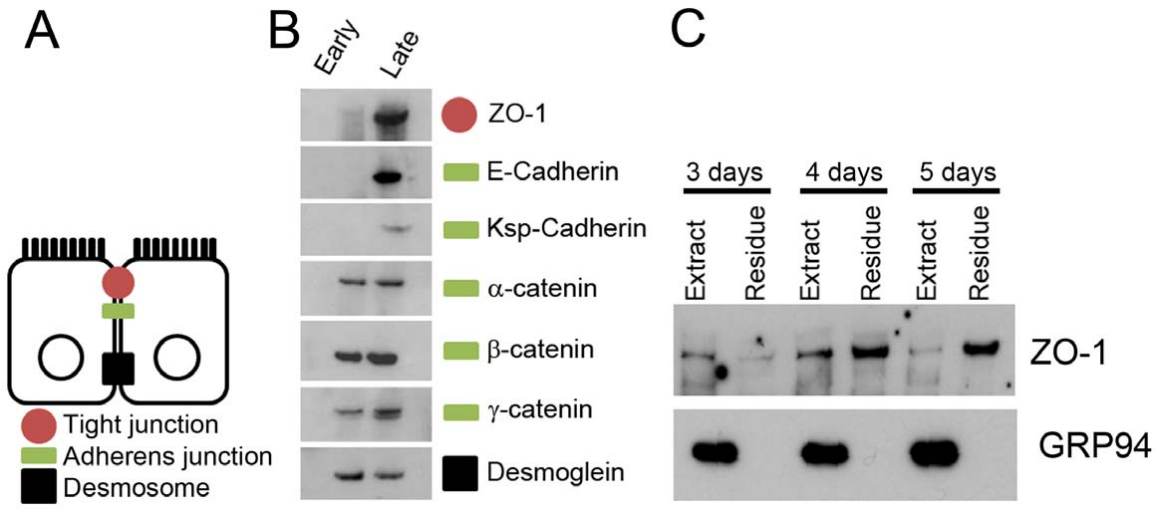
Maturation of the tight junction occurs as the nephron expresses markers of epithelium. A) A diagrammatic representation of the columnar epithelium of the proximal tubule. B) Western blot of early (0 day) and late (7 day) nephron cultures. Intercellular junction proteins specific to the tight and adherens junctions (Including the nephron specific KSP-Cadherin) denote the mesenchymal to epithelial transition during development of the nephron. C) Western blot showing detergent extraction of nephron cultures from 3 to 5 days. GRP94 was used as a control for extraction.

Central to this process of proximal tubular lineage determination is the mesenchymal to epithelial transition, establishment of apical-basolateral polarity, intercellular junctions and transporter function. In particular, the maturation of junctions such as the tight junction and the acquisition of functional transport capability in the developing proximal nephron has not been well defined in this model system. Therefore we examined these properties in the MM cultures as they developed into nephrons over the timeframe outlined above. As shown in Figure 1B, the epithelial markers ZO-1 (tight junction) and E-Cadherin (adherens junction) are expressed early in renal tubule development. To evaluate at what point in morphogenesis the nascent epithelium begins to establish mature junctions, proteins of the intercellular junctions were analyzed. Western blot analysis of the early and late MM cultures shows that intercellular junction proteins characteristic of epithelium are not present initially, but are expressed as the nephron develops (Figure 2B).

Although this data established that there was appropriate expression of intercellular junction proteins, it did not evaluate whether these proteins had yet associated with other proteins, such as those of the cytoskeleton; this process is necessary for full maturation and function of the tight junction upon which the paracellular barrier of the proximal tubule depends. Therefore, tight junction maturation was examined using a well-established detergent extraction method [34], in which molecules not anchored to the actin cytoskeleton (such as incompletely formed intercellular junctions) are extracted into a detergent-containing buffer while molecules closely associated with the actin cytoskeleton (including those in mature intercellular junctions) are inextractable and remain within the detergent-insoluble residue. Though widely used in cultured cells, to our knowledge, this has not been previously done in developing cultured MM; the expectation is that the fraction of inextractable tight junction protein increases with tubular development.

When MM was cultured for 3 to 5 days, tubular structures ranging from roughly S-shaped bodies to early tubules were observed (as shown in Figure 1C, D); the culture preparation was then extracted in a solution containing Triton X-100. The suspension was pelleted and western blot analysis of the pellet (residue fraction) or supernatant (extract fraction) was used to determine the extractability of the tight junction protein ZO-1. A shift in the ratio of extractable to inextractable ZO-1 occurred over that time, indicating the maturation of tight junctions, which, by this widely used biochemical criteria, at 5 days appear to have substantially matured (Figure 2C). Thus, even though significant tight junction protein expression is observed at 3 days, association with the cytoskeleton begins later (note the movement of more than half the amount of ZO-1 into the residue fraction at day 4) and is nearly complete at 5 days of culture.

### Genome Level Analysis of Developing Nephrons Highlights a Window for Proximal Tubular Differentiation

**Figure 3:**
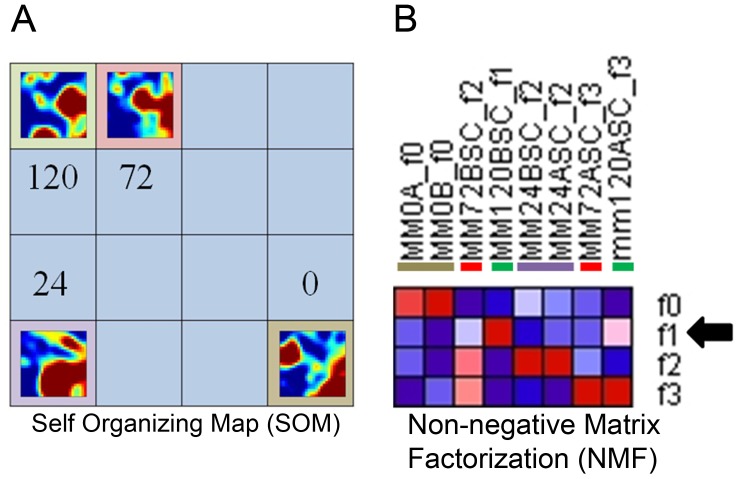
Informatic analysis of cultured nephrons highlights global stages of nephron development. A) self organizing map (SOM) featuring meta-meta representations of four points in nephron culture. Increasing distance between the samples on the map corresponds to increasing difference in the abstracted transcriptome. B) Non-negative matrix factorization (NMF) of the samples represented in panel A. The arrow highlights metagene F1, which comprises genes highly expressed only after 5 days (120 hours) in culture.

Published time-series microarray data from an in vitro nephron culture model was re-analyzed using several methods, including self-organizing maps (SOMs) [38] and non-negative matrix factorization (NMF) (Figure 3) [39]. SOM analysis identified changes in global gene expression between freshly isolated MM tissue and MM cultured for 1 day, a point at which epithelialization (MET) is beginning to occur (Figure 1A). A major difference is also observed between 1 day and 3 days. During this period the cultures initiate formation and maturation of nephron precursors, including comma-shaped, S-shaped and elongated S-shaped bodies (Figure 1B, C). A less drastic morphological difference was observed between three and five days of culture; however, analysis of the SOM heatmaps representing altered gene expression reveals that there are a number of genes which diverge between the two samples (Figure 3A). NMF was utilized to reduce the dimensionality of the data further, from 100 metagenes in the SOM to only 4 metagenes (Figure 3B). The NMF analysis indicated that one metagene (F1) comprises genes which are highly expressed at only 5 days of culture (when ZO1 is almost completely incorporated into the inextractable cytoskeletal fraction) but not at 3 days of culture (when ZO1 is almost fully extractable). These genes, mainly expressed after formation of elongated S-shaped bodies (morphologically the last step before formation of the proximal tubule and other nephron segments, and correlating to the time period in which tight junction maturation was shown to occur based on biochemical criteria), seem likely to include those involved in the later post-MET steps in the differentiation of the proximal tubule. Thus, in cultured nephrons, biochemical and informatic analyses identify a time frame in which the earliest signs of this nephron segment differentiation are established.

### Microarray Time Series Analysis of In Vivo and Ex Vivo Development of Nephrons Enables Creation of a Preliminary Network for Proximal Tubulogenesis

Having established a window for the maturation and differentiation of the proximal tubule (between 3 and 5 days of culture), a global approach to identifying the genetic mechanisms regulating the functional maturation process was undertaken. Several genes known to be contributors to the process of nephron segmentation are expressed at the S-shaped body stage of development, and ultimately affect the differentiation of the nephron. For instance, genetic deletion of the Brn1 gene causes a disruption in the differentiation of the Loop of Henle and the distal tubule, while Notch2 is required for proximal tubule fate [17], [30]. These genes, however, are two of only a few known genes that control nephron segment differentiation. While genes already known to play a key role in nephrogenesis do not alone provide new information about the differentiation of the proximal tubule, an expanded developmental program with potentially novel and important connections begins to emerge with the addition of potential interactors from relevant transcriptomic analyses.

To move towards a transcriptome-wide understanding of the process of proximal tubule differentiation, microarray data from in vivo and ex vivo nephrons was analyzed. Analysis of genes expressed in the developing nephron was done at two stages corresponding approximately to the s-shaped body and early proximal tubule (based on the biochemical data described above). Those genes which significantly differed between 3 and 5 days in cultured MM were used as a set of potential interactions, since they correspond temporally to structural and marker-based nephron development and biochemically-defined tight junction maturation and functional transport of organic anions by the developing nephron (see above). In order to ground the ex vivo data to an in vivo dataset, microarray data was downloaded from the GUDMAP database for the mouse e15.5 s-shaped body and early proximal tubules. This data was analyzed for differentially expressed genes, and the intersecting genes between the in vivo and ex vivo analyses were used to build a network of genes representing interactions during proximal tubule development and maturation. Analyzing the data in this manner would thus help establish in vivo relevance to the analysis that follows.

**Figure 4:**
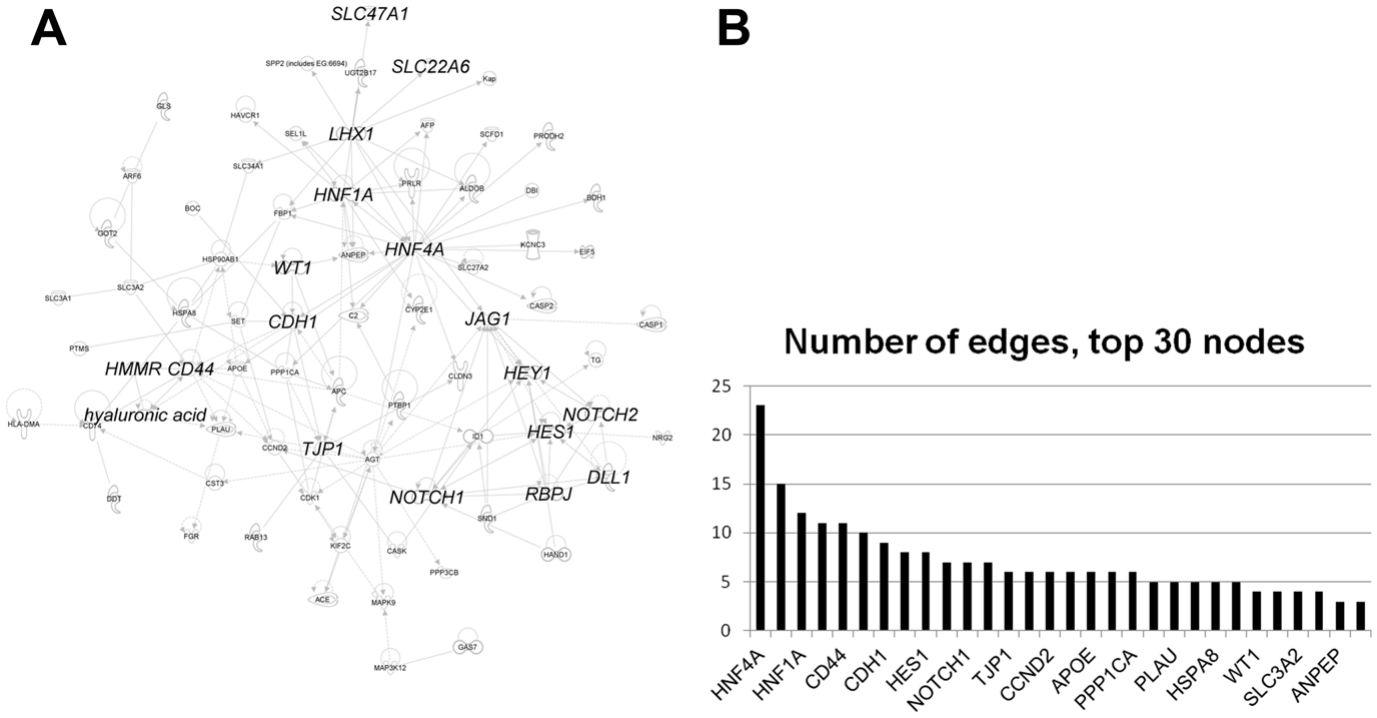
Putative proximal tubulogenesis gene network shows high connectivity of Hnf4α. A) The genes that significantly differ from 72 to 120 hours in ex vivo nephron culture and from S-shaped body to proximal tubule in vivo represented in an interaction network based on the proximal tubule “legacy” genes shown in Table 1. B) Quantitative representation of the number of connections (edges) each gene (node) has to others in the proximal tubulogenesis network shown in panel A. Hnf4α is the most influential (largest number of edges) node in the network.

As a starting point to anchor the genes arrived at statistically, a “legacy” list of genes that interact with or are known regulators of nephron development and core function were used as the seed genes for network generation (Table 1). The legacy list of genes is based on many different studies detailing the development and function of the proximal tubule. The genes include cell adhesion molecules, surface receptors, signaling pathways and transcription factors, and clinically relevant proximal tubule transporters. This “legacy” network can be viewed as the ground upon which the analysis below was carried out. Using Ingenuity Pathways Analysis (IPA), the “legacy” network was connected to the genes that significantly differ in the in vivo and ex vivo s-shaped bodies and early proximal tubules (Figure 4A).

**Table 1:** Literature based “legacy” genes provide a core to build a SS to PT development network.

Gene (Node)	Source
Notch 1, 2	[17], [21]
RBPJ	[21]
Hyaluronic Acid	[26]
CD44 (HA receptor)	[26]
RHAMM (HMMR, HA receptor)	[26]
E-Cadherin (CDH1)	[27]
ZO-1 (TJP1)	[27]
Lim1 (LHX1)	[29]
Brn1 (POU3F3)*	[30]
Oat1 (SLC22A6)	[12]
Oat3 (SLC22A8)	[12]
Oct1 (SLC22A1)	[12]
Oct2 (SLC 22A2)	[13]
(SLC 47A1)	[13]
(SLC 15A2)	[13]
WT1	[28]
Jag1	[17]
Dll1	[17]
Hey1	[22]
Hes1	[22], [23]
Hnf1a	[24]
Hnf4a	[25]

Genes representing known regulators of nephron development and of proximal tubule function were used as the basis of the s-shaped body to proximal tubule stage specific network. This core of genes is the basis for addition of differentially expressed genes from in vitro and in vivo microarray experiments.

* Brn1 is determinant of distal tubular development.

The nodes in the network comprise a diverse set of molecules, including many genes of the functional proximal tubule and included multiple SLC family molecular transporters, as well as expected xenobiotic metabolizing enzymes. For instance, the Na/Pi co-transporter (Slc34A1) is a well-known the proximal tubule gene. Interestingly, the network included two markers of proximal tubular injury, Kim1 (HAVCR1) and Cystatin C (CST3) [40].

Furthermore, as the number of studies utilizing microarray experiments increases, the results obtained from independent analyses of the data can be utilized to augment the results of other analyses of the data. For example, recently a number of genes identified from the same in vivo microarrays analyzed above were verified to be explicitly expressed in the early proximal tubule [25]. Where this list of genes was non-overlapping with our statistical analysis, the genes were added to the network. Finally, the nodes that did not connect to any others were removed from the network for clarity.

### Pathway Model Regulating Proximal Tubulogenesis and Organic Anion Transport Function: A Potential Role of Hnf4α

To begin to examine the origin of the emergence of proximal tubular function, we analyzed microarray data chronicling the developing nephron, in vivo and ex vivo. The molecule that illustrated the highest connectivity was Hnf4α, a known regulator of nephrogenesis (Figure 4B). Although Hnf4α has been studied in detail in other organs [41], [42], [43], [44], and there is data suggesting a role in early development [45], it remained to be determined whether or not Hnf4α was contributing to the gain of proximal tubular capacity to transport organic anions and organic cations (through Oat1, Oat3 and Oct1). These transporters are known to be crucial to the mature function of the proximal nephron, although Oct1 and Oat3 did not connect to other nodes in the network.

Oat1 and Oat3, the prototypical organic anion transporters of the nephron, are responsible for handling many drugs, toxins, metabolites, and commonly used anti-virals [46], [47], [48]. Deletion of Oat1 in mice results in the accumulation of many endogenous organic anion metabolites [5], a diminished diuretic response and protection of the kidney from mercury toxicity [8]. Oat3 knockout mice also have transport defects, and exhibit cross-specificity with many Oat1 substrates [9]. Oct1, similarly, is a multispecific transporter of cations which, when combined with null mutation in Oct2 (Slc22A2), greatly diminishes the ability to excrete prototypical cations through the proximal tubule [49]. Polymorphisms in Oct1 are associated with drug toxicity from metformin [50].

**Figure 5:**
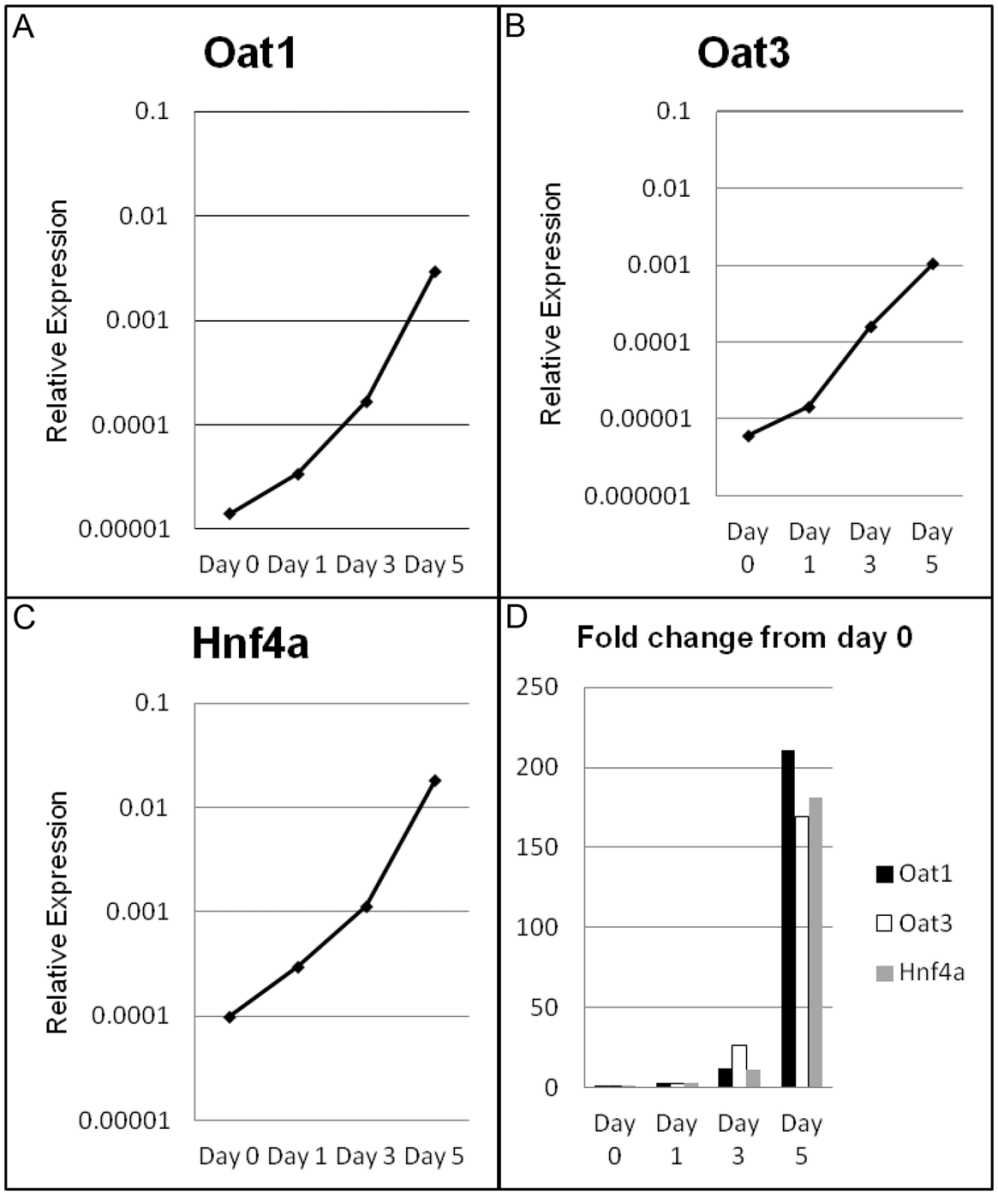
qPCR validation of Oat and Hnf4α expression in cultured nephrons. Expression of Oat1 (A), Oat3 (B), and Hnf4α (C) show change in expression in developing nephrons and are expressed as normalized expression, relative to GAPDH. D) The fold change in each transcript.

Hnf4α is highly expressed in the developmental transition from s-shaped body to early tubule, when the tight junction appears to mature and the tissue is capable of Oat mediated transport. A time series of cultured nephrons were analyzed by qPCR for expression of Hnf4α. The expression of Hnf4α increases over culture time, with a rapid and dramatic increase (approximately 200 fold change) seen from 3 to 5 days in culture (Figure 5C), the proximal tubule maturation/differentiation “window” established above by the global profiling and biochemical analysis of tight junction proteins. A similar profile and magnitude of expression was observed in the transcription of Oat1 and Oat3 (Figure 5A, B). Thus, the temporal analysis described so far suggests that the proximal tubule requires sequential tight junction maturation (as measured by detergent extractability), followed by Oat gene expression, and this correlates with Hnf4α expression. All of this led us to hypothesize a key role for Hnf4α in Oat and Oct expression during proximal tubule maturation, despite the lack of a direct link in pathway analysis.

**Figure 6:**
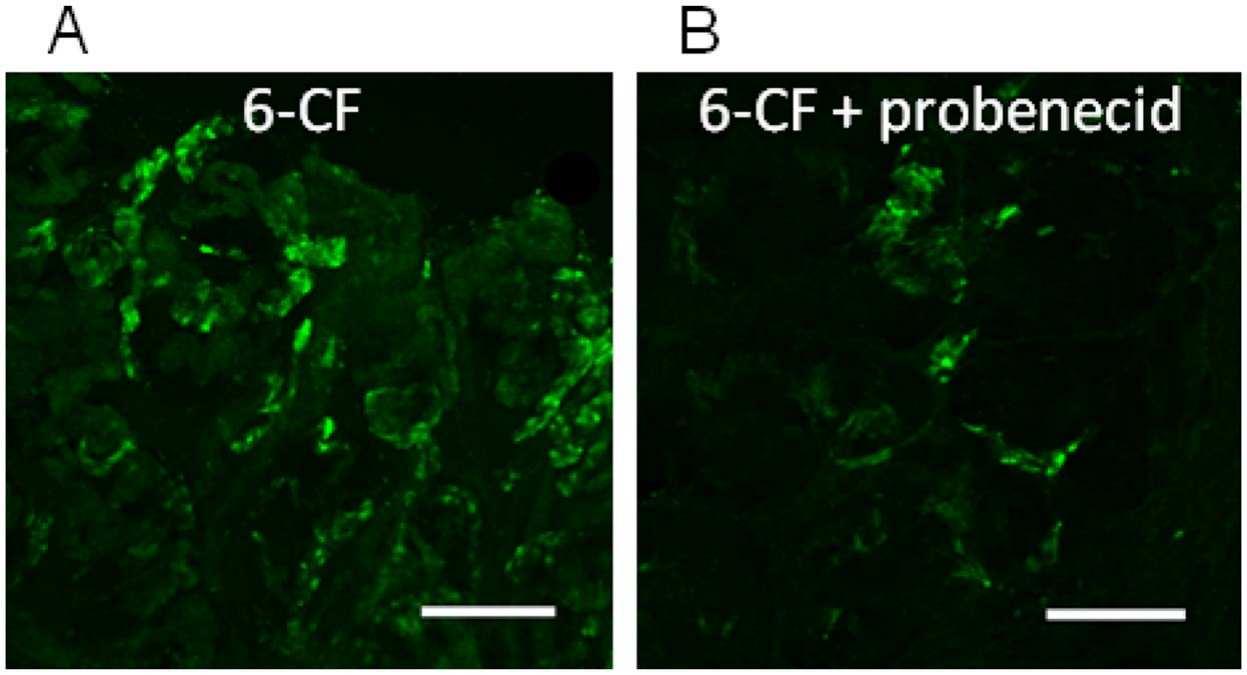
Functional transport occurs simultaneously with tight junction maturation and Oat expression. A) Immunofluorescent image of late nephron cultures incubated with the Oat-selective organic anion 6-carboxy fluorescein (6-cf). B) Immunofluorescent image of late nephron culture incubated with 6-cf and the Oat inhibitor probenecid. The bar (A and B) is 100 µm.

During the transition from early to late nephron, coinciding with increasing expression of Hnf4α, Oat1 and Oat3, the nascent tubules are generally thought to be capable of preventing paracellular transport of organic anions once the functional tight junction is established. A strong increase in Oat expression was seen to coincide with detergent inextractability of ZO1 (Figure 2C). MM culture expression analysis of Oat1 and Oat3, which mediate metabolite, drug and toxin handling [1] revealed an increase of transporter expression over time in culture [18], consistent with previously published in vivo time series data [13]. To functionally test organic anion transport during ex vivo tubular nephron formation, the capacity to handle an Oat specific organic anion tracer was analyzed in late cultures which have not undergone complete nephron segmentation or full nephron differentiation (Figure 6). The tracer molecule, 6-carboxy-fluorescein (6CF), was internalized under standard assay conditions. Moreover, this effect was inhibited in the presence of the oat inhibitor, probenecid, suggesting the presence of functional organic anion transport capacity in the pre-natal proximal tubule, concordant with published gene expression data [13]. Thus, it appears that some degree of organic anion transport by Oats can occur prior to complete nephron segmentation and postnatal maturation concomitant with the establishment of the mature tight junction.

### In Silico Promoter Analyses of SLC22 Family Transporters Predicts Regulation by HNF4α

**Figure 7:**
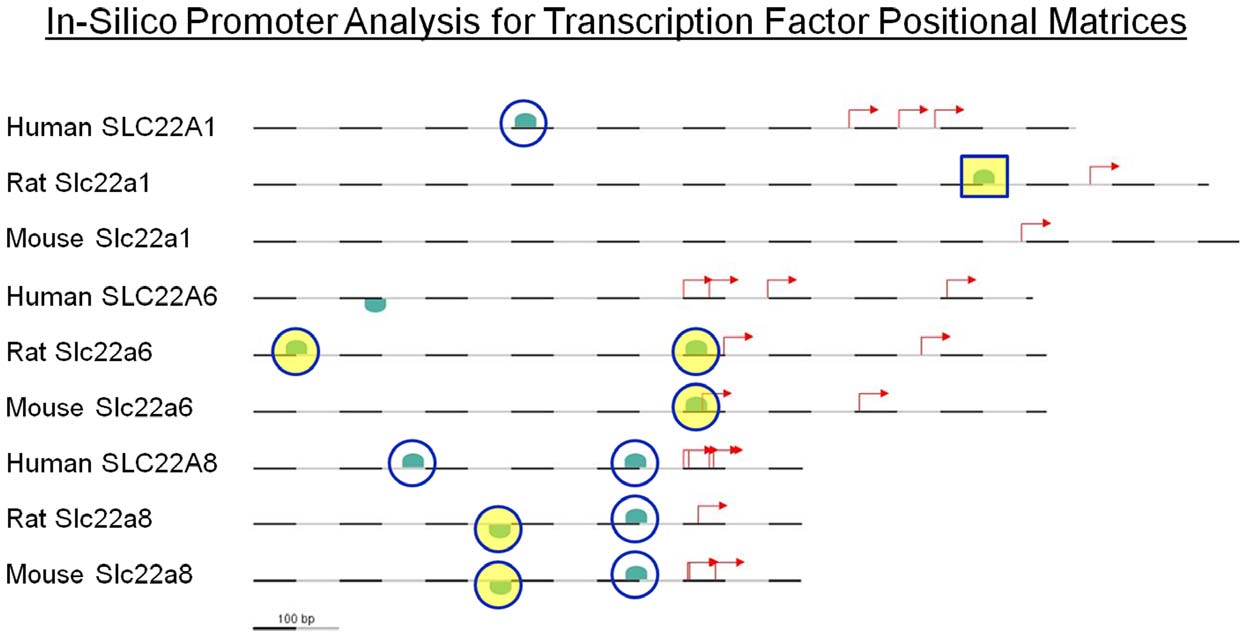
In silico promoter analysis of Oat1, Oat3, and Oct1 transporters predicts regulation by HNF4α. Diagram of the promoters of the human, rat, and mouse Oct1, Oat1 and Oat3 promoters. Under stringent settings only a single TRANSFAC binding motif remained - V$NR2F. Six transcripts had a DR-1 binding element within the proximal promoter region, and one had a DR-2 binding element, resulting in 7 of 9 transcripts predicted to have the potential to recruit Hnf4α directly. Green markers depict binding sites for members of the Nuclear Receptor 2 family as a function of position relevant to known transcription start sites. DR-1 elements are circled, the single DR-2 element is marked by a square. Yellow shading denotes a direct call of HNF4α by Genomatix.

Analysis of phylogenetic footprints of human and mouse Oat1 and Oat3 promoters has shown binding sites for known regulators of renal development, including WT1 and HNF1 [51]. Because Oct1, Oat1, and Oat3 are representative of proximal tubule identity, we decided to use Genomatix Software Suite to look for cis-regulatory elements in proximal promoter regions of the most significant transcripts for these genes (Figure 7). High stringency analysis resulted in the predicted functional binding sites to come from a single group – the Nuclear Receptor 2 Family, which includes Hnf4α. Five out of the nine transcripts analyzed were found to have sites that were called as Hnf4α sites, while seven of the nine had sites that potentially bound HNF4α.

The expression and biochemical data presented above suggest that there is simultaneous expression of Hnf4α and Slc22 family transporters in the developing proximal tubule. Specific proximal tubular expression and function of the Slc22 family of transporters, along with Hnf transcription factors is well documented in the literature (summarized in Table 2) [5], [9], [13], [20], [52], [53], [54], [55], [56], [57]. Further, in silico and in vitro evidence links the Hnf transcription factors to Oct1, Oat1, and Oat3 (summarized in Table 3) [51], [58], [59], [60]. The in silico analysis of these promoters (Figure 7) predicts that Hnf4α acts upon these promoters during the maturation of the proximal tubule. The relationship between Hnf4α and these three proximal tubule transporters has not been directly observed in vivo during proximal tubular maturation. The evidence justified examining whether these predicted regulatory sites are actually occupied by Hnf4α in-vivo.

**Table 2:** Expression and protein localization of Oat1, Oat3, Oct1, and Hnf4α in the proximal tubule.

Gene	Expression in PT	Protein localized to PT	Renal function through knockout
Oat1	Microarray [13]	IHC [53]	[5]
	Microarray, ISH [20]	IHC [52]	
Oat3	Microarray [13]	IHC [53]	[9]
		IHC [52]	
Oct1	Microarray [20]	IHC [54]	[57]
	mRNA expression (Whole kidney) [55]		
	ISH [54]		
Hnf4α	Microarray [20]	IHC [56]	
	ISH [56]		

Evidence from existing literature validates the result of microarray analysis. The genes and protein products of Hnf4α, Oat1, Oat3, and Oct1 are present in the proximal tubule during organogenesis and adulthood.

**Table 3:** Hepatocyte nuclear factors are associated with Slc22 genes in silico and in vitro.

Gene	In vitro or in silico experiment
Oat1	Promoter analysis [51]
	In vitro regulation [58]
	In vitro regulation [59]
Oat3	Promoter analysis [51]
Oct1	Promoter analysis, in vitro regulation [60]

Evidence from the literature demonstrates the potential for functional regulation of the SLC22 family of genes by hepatocyte nuclear factors.

### Hnf4α Occupies the Oat1 and Oat3 Promoter Regions In Vivo

Because Oct1, Oat1, and Oat3 are representative of proximal tubule identity, a direct link was sought between Hnf4α and Oat expression in the maturing nephron. While multiple mouse models have been created to investigate Hnf4α function, the contribution of Hnf4α in the proximal tubule cannot simply be inferred based on existing lines. The Hnf4α null mutant undergoes impaired gastrulation leading to embryonic lethality [45], which has necessitated tissue-specific knockouts. Interestingly, conditional mutants have revealed surprisingly diverse context specific roles for Hnf4α. In the liver, Hnf4α is required for MET and hepatocyte differentiation, thus resulting in severe dysgenesis in its absence [41]. Conditional deletion of Hnf4α in the embryonic colon also revealed a role in epithelialization and a requirement for normal development. However, deletion in the colon upon epithelialization was mainly characterized by dysregulation of ion transport [42]. Surprisingly, while the most significant effect of heterozygous Hnf4α mutations in humans is B-islet dysfunction, and Hnf4α function is thought to be highly conserved between rodents and humans [44], conditional deletion in the pancreas does not perturb morphogenesis [43]. While in vitro approaches have suggested a role for Hnf4α in condensed mesenchyme maintenance and early epithelialization in the kidney, the role of Hnf4α in proximal tubule maturation/differentiation has not been defined [61], [62]. Moreover, although this gene has not been specifically deleted in the kidney, the in vitro data suggests that it may be difficult to analyze late differentiation in the proximal tubule owing to its role in mesenchyme maintenance and early epithelialization. There does not appear to be a satisfactory construct to specifically knock out the gene in the equivalent of the day 3 to day 5 “window” of the MM culture system that we have identified as critical.

Nevertheless, promoter region analysis of the Oat1, Oat3 and Oct1 genes suggests Hnf4α binding sites. We therefore assayed Hnf4α regulation of SLC22 family transporters in in vivo nephron maturation by determining whether Hnf4α occupies the promoters of the Oat1, Oat3, and Oct1 genes. This was examined in vivo in neonatal rat kidneys (in which proximal tubule maturation is ongoing) using chromatin immuno-precipitation (ChIP) followed by qPCR. The immature rat kidney was examined after the end of tubulogenesis, but before proximal tubules reach maturity, at approximately 3–4 weeks after birth [13]. Hnf4α was found to occupy the promoters of the Oat1, Oat3, and Oct1 genes compared to two negative control regions (Figure 7). In the kidney, Hnf4α expression is known to be restricted to the proximal tubules after nephrogenesis [20], [25], [56], and thus enrichment potentially indicates Hnf4α involvement in transcriptional regulation of Oat1, Oat3, and Oct1 in the proximal tubule in vivo.

## Discussion

Here we have used a combination of bioinformatic, biochemical and functional approaches to show that there is initial proximal tubule segment maturation in the late cultured nephron (Figure 1E) and link the onset of functional tubular cells to Hnf4α. The maturation of the tubular tight junction corresponds to the rise in Oat expression in the system (Figures 2C and 5). At this time, the tubule engages in Oat-mediated vectorial transport of organic anions, which is suppressed by the classical Oat inhibitor, probenecid (Figure 6). This occurs despite the fact that nephron segmentation is incomplete. Microarray analyses of ex vivo and in vivo samples containing S-shaped bodies to early proximal tubules led us to hypothesize a role for Hnf4α in the functional maturation of the proximal tubule. ChIP-qPCR was used to confirm Hnf4α occupancy at promoters of characteristic transporters (Oat1, Oat3 and Oct1) during proximal tubule maturation, further supporting the involvement of Hnf4α in the transcriptional regulation of proximal tubular functional characteristics.

It was found that the epithelial tubule of the nascent nephron begins to develop functional characteristics before full segmentation of the nephron has occurred. Specifically, prior to reaching maturation, nascent nephrogenic structures are capable of organic anion transport. Interestingly, while stimuli are provided by an inductive tissue source, the initiation of specific transport functionality does not require the presence of a collecting system, the in vivo source of the nephrogenic stimulus. It may be important that this process occurs early in development, as this may enable the fetus to cope with endogenous toxins or transport key metabolites and small molecule morphogens in the early stages of nephrogenesis [47], [63]. In the case of humans, it may be important for the movement of certain pharmacologic treatments to a, newborn or in preterm survival when infants are born before nephrogenesis is complete.

Due to its degree of connectivity to genes whose expression increases from 3 to 5 days and from S-shaped body to early proximal tubule, it is likely that Hnf4α plays a key role in the functional differentiation of the renal tubule (Figure 4). It has documented effects on the morphogenesis and function in other epithelial tissues (liver, intestine, and pancreas), but has not been demonstrated during renal organogenesis [42], [64]. Interestingly, Hnf4α appears to affect nephron development in distinct phases, at the MET and at functional maturation. Our network identifies separations from Hnf4α to other known pathways affecting PT production, including Notch and hyaluronan signaling, which have been linked to proximal tubule growth and differentiation [17], [21], [26]. Additional evidence from nephron development may suggest specific genetic environments leading to regulation of basic epithelial characteristics versus tissue-specific, or mature, characteristics.

Previous studies have shown that Hnf4α plays a role in regulating many of the functional properties attributed to these genes in other epithelial tissues. In the colon, liver, intestine and pancreas, Hnf4α appears to be involved in the regulation of transport, metabolism and epithelial properties, all of which are critical for proper function of the proximal tubule [64]. Our data suggests Hnf4α may potentially play a large role in defining the cellular identity of proximal tubule cells as well. The activity of Hnf4α or Hnf1α (a co-regulator of SLC family transport expression) on Oat expression has been implicated through promoter analysis and in vitro experimentation [51], [58], [59], [65], [66]. This new in vivo evidence demonstrates binding of Hnf4α onto the promoters of these key proximal tubule genes during a period of increasing renal differentiation, timed appropriately with recently demonstrated localization of Oat proteins along the rat proximal tubule [13], [52].

**Figure 8:**
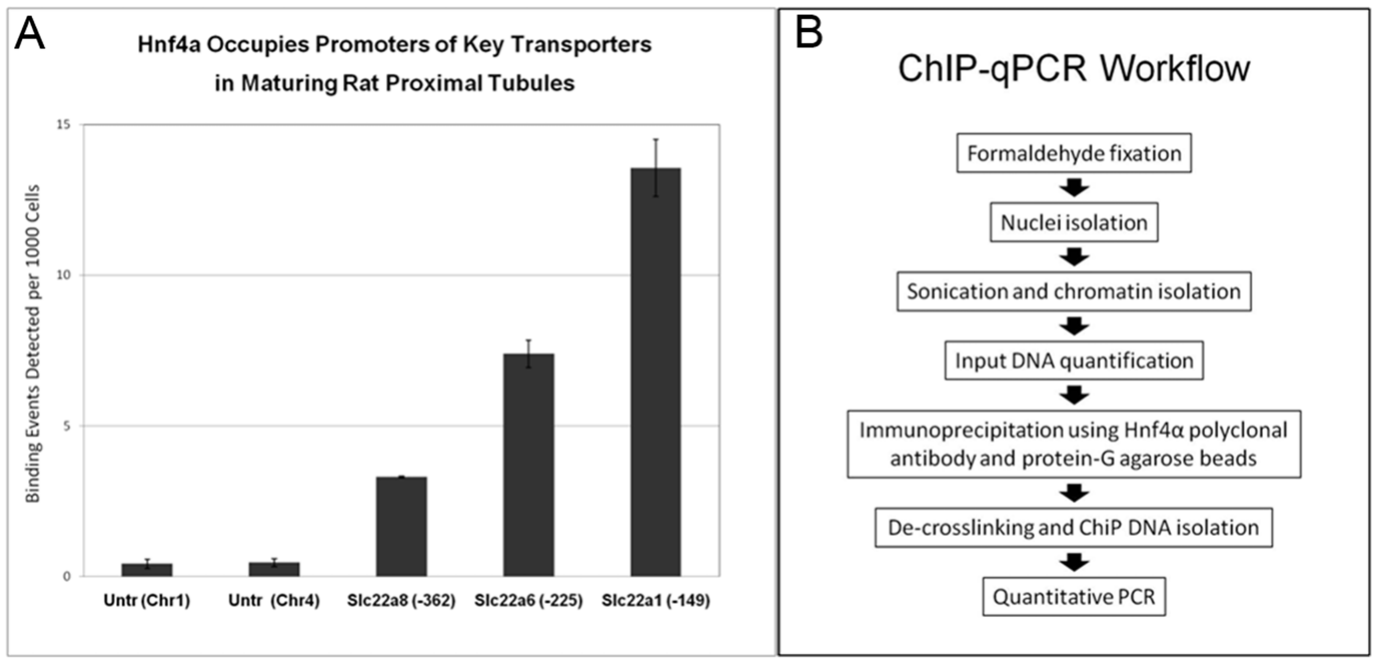
ChIP-qPCR confirms Hnf4α binds the proximal promoters of transporters in the in vivo maturing nephron. A) ChIP qPCR of Oct1 (Slc22a1), Oat1 (Slc22a6) and Oat3 (Slc22a8) promoters using an HNF4α antibody. Two loci outside of annotated coding or transcribed regions on chromosome 1 and 4 were used as negative controls. B) Schematic workflow of the chromatin immunoprecipitation qPCR experiment.

The prediction of high connectivity of Hnf4α with genes thought to be involved in proximal tubule morphogenesis and maturation, with the finding that it is highly enriched at the promoters of prototypical drug-handling transporter genes (Oat1, Oat3, Oct1 in Figure 8), supports its importance in the emergence of proximal tubular functional characteristics. This data is in agreement with the bioinformatics analysis of genes with expression restricted to the nascent proximal tubule [20].

The finding that kidney function may be heavily regulated at the transcriptional level by a ligand-dependent nuclear receptor raises the prospect of developing pharmacotherapies aimed at enhancing or protecting kidney function in premature, injured or aging kidneys. In fact, Hnf4α-mediated transcription is susceptible to a multitude of signaling mechanisms [67], thus increasing the likelihood that pharmaceutical intervention may be beneficial to counteract undesirable fluctuations, or to enhance function when necessary. As with other physiological functions involved in homeostasis, renal toxin clearance is likely to remain under dynamic regulation after development with Hnf4α acting as a key site of regulation in the proximal tubule and potentially as a sensor in the hypothesized remote sensing and signaling network involving SLC22 and other xenobiotic transporters [10], [11].

Based upon the current ChIP-qPCR data, Hnf4α is directly linked as a transcriptional regulator of Oat1, Oat3, and Oct1, the primary drug-handling transporters of the kidney. This links the process of functional drug excretion in the kidney to a well-established developmental mechanism in other tissues. While a single transcription factor cannot be solely responsible for all specific cellular characteristics, studies have shown that a small group of transcription factors is often responsible for a majority of specific lineage traits [68], [69]. Further understanding of the role Hnf4α plays in proximal tubule differentiation, maturation and should not only enhance the understanding of late kidney development but suggest approaches to modulating the expression of Oats and other SLC22 transporters in order to facilitate handling of toxins or drugs in disease settings [6], [8].
